# Short-Term Traffic State Prediction Based on the Spatiotemporal Features of Critical Road Sections

**DOI:** 10.3390/s18072287

**Published:** 2018-07-14

**Authors:** Gang Yang, Yunpeng Wang, Haiyang Yu, Yilong Ren, Jindong Xie

**Affiliations:** 1 School of Transportation Science and Engineering, Beijing Key Laboratory for Cooperative Vehicle Infrastructure System and Safety Control, Beihang University, Beijing 100191, China; yanggang@buaa.edu.cn (G.Y.); ypwang@buaa.edu.cn (Y.W); yilongren@buaa.edu.cn (Y.R.); 2 Beijing Advanced Innovation Center for Big Data and Brain Computing, Beihang University, Beijing 100191, China; 3 Jiangsu Province Collaborative Innovation Center of Modern Urban Traffic Technologies, Sipailou 2, Nanjing 210096, China; 4 School of Electronic and Information Engineering, Beihang University, Beijing 100191, China; jindong.xie@buaa.edu.cn

**Keywords:** short-term traffic prediction, structural missing data, deep learning, critical road sections, spatiotemporal correlation

## Abstract

Recently, short-term traffic prediction under conditions with corrupted or missing data has become a popular topic. Since a road section has predictive power regarding the adjacent roads at a specific location, this paper proposes a novel hybrid convolutional long short-term memory neural network model based on critical road sections (CRS-ConvLSTM NN) to predict the traffic evolution of global networks. The critical road sections that have the most powerful impact on the subnetwork are identified by a spatiotemporal correlation algorithm. Subsequently, the traffic speed of the critical road sections is used as the input to the ConvLSTM to predict the future traffic states of the entire network. The experimental results from a Beijing traffic network indicate that the CRS-ConvLSTM outperforms prevailing deep learning (DL) approaches for cases that consider critical road sections and the results validate the capability and generalizability of the model when predicting with different numbers of critical road sections.

## 1. Introduction

Real-time traffic state prediction plays a vital role in traffic management and public service. The ability to timely, accurately and efficiently predict the evolution of traffic can assist travelers and government agencies in reacting to possible congestion ahead of time. In recent decades, reliance on efficient access to massive traffic data, such as loop detectors and floating cars carrying global positioning system (GPS) devices, has provided an impetus for a considerable amount of data-driven computational approaches to estimate and forecast future traffic states. Compared with data-driven methods, mathematical or statistical models that are derived from macroscopic and microscopic theories of traffic flow exhibit difficulties addressing unstable traffic conditions and complex road settings due to their strong hypotheses and assumptions [1].

Data-driven methods, including the autoregressive integrated moving average model (ARIMA), support vector machine (SVM), Bayesian network, neural network (NN) and so forth, have achieved promising results due to their greater potential in processing complex nonlinear problems [2]. Among all the data-driven methods, the deep learning approach has been validated for its efficiency since it is capable of exploiting much deeper architectures and processing high-dimensional sets of explanatory variables in transportation networks [3]. However, the veracity of the data (i.e., noisy and missing data) in the real world affects predictive accuracy to a great extent. Especially for taxi-based floating car data (FCD), spatial coverage relies heavily on the penetration rate of probing taxis [4]. The use of lower density probes on certain roads will result in a large variance of traffic speed that does not accurately reflect real traffic conditions. This problem often occurs on minor roads and leads to structurally unreliable traffic data. In addition, urban tall buildings and other large objects usually block or strongly degrade GPS signals to invalidate road traffic data [5]. These setbacks caused a long-term loss of traffic data on some road sections, namely structural missing data, which weakens the predictive accuracy and limits the ability to provide a practical and reliable forecasting result. This paper seeks to provide a solution to settle these matters.

The influence between downstream and upstream areas has been studied for a long period of time [6]. Recently, researchers have found that previous conditions of a road section have an impact on the current state of its adjacent roads at a specific local network [7]. This conclusion indicates that the future traffic conditions of a road can be predicted by the current state of its neighbors. The predictive power between two adjacent roads can be estimated by their spatiotemporal correlation [8]. Therefore, if we extract data on the critical road sections that have the most dominant predictive power, we can characterize the spatiotemporal features of traffic flow and predict the future traffic conditions of the overall network. In this way, the traffic conditions of minor roads affected by structural missing or unreliable data can be predicted by their critical adjacent roads.

In this paper, we propose a novel hybrid convolutional long short-term memory neural network model based on critical road sections (CRS-ConvLSTM NN) to predict future traffic conditions in the overall network. With the assistance of a spatiotemporal correlation algorithm (STCA), the critical road sections that have a significant impact on the local traffic networks are identified and extracted from the network. First, the spatial relevance among road sections is evaluated by establishing a spatial adjacency matrix. The matrix is constructed according to the topological relationships of the urban network. Then, the strength of the influence between a given road section and its neighbors under conditions of a 2 min to 1 h time delay can be calculated based on the correlation theory. The degree of influence can be adopted as a criterion for evaluating the importance of the roads. Because the value of the spatiotemporal correlation is different for each delay of each road section, the technique for order preference by similarity to an ideal solution (TOPSIS) is applied to synthetically consider its impact on the subnetwork by ranking their orders. Then, a certain proportion of road sections is regarded as the critical sections according to their ranks.

In the prediction section, we introduce the ConvLSTM NN and show that it outperforms other models, such as artificial neural networks (ANNs), convolutional neural networks (CNNs), long short-term memory neural networks (LSTMs) and the stacked autoencoder model (SAE). The number of critical road sections we extracted from all the road sections affects the forecast performance in terms of the loss function of the CRS-ConvLSTM model. To the best of our knowledge, this paper presents the first report of a method for predicting the urban traffic evolution on a network-wide scale with partial sections that characterize the spatiotemporal features of the integral network.

The contribution of the CRS-ConvLSTM model lies in addressing structural data failure problems by exploiting the potential of the predictive power among sections. With the preprocessing strategies exerted on the input data based on the STCA, our model can clearly reflect the spatial and temporal characteristics of the road network. Practical experiments on real-life speed data in a large-scale network of Beijing City demonstrate that the proposed CRS-ConvLSTM model can precisely predict the future traffic network states. Additionally, the fluctuation of performance under different numbers of critical road sections and various situations (e.g., stochastic and extreme case) is also tested.

The remainder of our paper is outlined as follows. Section 2 discusses the literature on real-time short-term traffic prediction. Section 3 elaborates on the spatiotemporal correlation algorithm to determine the degree of impact of the road sections and proposes a method of identifying the critical road sections. Section 4 constructs the CRS-ConvLSTM model to predict the speed of the overall traffic network based on the data of critical road sections. To validate the effectiveness of the proposed approach, experiments on a large-scale traffic network of Beijing City are conducted. Section 5 evaluate the performances under different numbers of critical road sections based on the predictive accuracy. The final section presents the conclusions and discusses future work.

## 2. Literature Review

In recent decades, a considerable number of short-term traffic forecast models have been developed to predict future traffic states ranging from a few seconds to a few hours ahead. The approaches can be generally categorized into parametric and nonparametric approaches [3,9]. The parametric approaches mainly focus on predetermining the structure of the model based on theoretical or physical assumptions and then tuning a set of parameters to faithfully reflect the evolution of traffic conditions in the real world [3,9]. Among all the parametric approaches, model-based time-series analyses, especially ARIMA, are widely used for traffic predictions. Since Ahmed and Cook first introduced the ARIMA model in univariate single-point settings [10], a number of variations, such as seasonal ARIMA [11] and STARIMA [12], were proposed to improve prediction accuracy. However, the majority of parametric approaches fail to provide a favorable result under unstable traffic conditions, such as unexpected events or extreme weather [3]. To address this problem, numerous nonparametric approaches have been proposed and they present a number of advantages, such as the ability to be free from strong assumptions underlying the model and to adaptively learn the implicit dynamic traffic characteristics through archived traffic data; these approaches include Kalman filters [13,14,15], SVM [16,17,18], NNs [19,20,21,22] and hybrid methods [23,24].

Vlahogianni et al. indicated that artificial intelligence (AI) has gradually become the dominant approach for transportation prediction because of its inherent propensity for hypothesis-free techniques that are capable of addressing multidimensional and nonlinear problems [3]. With the strength of flexibility, adaptability, learning and generalizability, NNs have received tremendous interest and been extensively used as a capable alternative to address transportation problems. Smith and Demetsky established back-propagation neural networks (BPNNs) to forecast traffic volume and the results clearly showed that the BPNNs outperformed the historical average and ARIMA models [21]. Chen and Grant-Muller investigated the dynamic characteristics of a neural network and constructed dynamic NNs in cooperation with varied and complex traffic patterns [25]. Huang et al. constructed a NNs model to predict traffic speed by considering weather conditions, specifically the visibility, temperature and moisture [26]. Another line of research on short-term traffic prediction incorporated traditional NN approaches with other computational intelligence (CI) approaches or statistical methods since the prediction that depended exclusively on NNs lacked an optimal generalization capability [27]. Chan et al. adopted a hybrid exponential smoothing method and Levenberg-Marquardt algorithm (EXP-LM) to remove the lumpiness that weakens the generalization capability of the approach from traffic data and then trained the model with NNs [27]. With the purpose of avoiding arbitrariness when selecting a suitable structure of a network, Hinsbergen et al. calculated an evidence factor based on a Bayesian committee to facilitate the integration of different NNs and it obviously outperformed a single NN when applied to an empirical experiment [28].

Recently, deep learning approaches capable of processing nonlinear high-dimensional problems have been increasingly developed; these approaches are capable of addressing complex traffic flow characterized by both spatial and temporal features. Different from other traditional ANNs models, deep learning algorithms extract inherent features of the data using multiple layers or deep architectures and thus achieve better results [29]. Under the guidance of applications developed in the domains of image, video, audio and language learning tasks [30], deep learning approaches have proven their effectiveness for traffic predictions. In 2014, Huang et al. employed a deep belief network (DBN) with multitask learning for traffic flow predictions [2]. Lv et al. proposed an SAE and its performance is superior to that of BPNNs, the random walk (RW) forecast method, SVMs and the radial basis function (RBF) NN at different prediction horizons [31]. Furthermore, the recurrent neural network (RNN), which presents the intrinsic properties of creating and processing memories of arbitrary sequences of input patterns, is regarded as an excellent model for capturing the time sequential features of traffic flow, especially the LSTM network because it overcomes the vanishing and exploding gradient problems when capturing long temporal dependency [32,33,34,35]. Another promising branch of NNs that has been widely adopted for traffic forecasting is the CNN. Ma et al. viewed the traffic conditions as sequential images and then predicted traffic speeds after 10 min and 20 min based on the spatiotemporal features of the network [36].

Furthermore, numerous efforts have been devoted to emphasizing the temporal characteristics and spatial dependencies of predictions [3]. Min et al. proposed a multivariate spatial-temporal autoregressive (MSTAR) model that defined spatial correlations among links by the reachability of one link was reachable by another in a certain time interval [8]. Cai et al. predicted multistep traffic conditions using a k-nearest neighbor model based on a spatiotemporal state matrix [37]. Habtemichael et al. predicted future traffic through an enhanced K-NN method. A similar historical dataset was identified as the forecasting reference by means of the weighted Euclidean distance and the rank-exponent method [38]. In recent years, deep learning approaches, especially CNNs and LSTMs, have contributed to capturing spatiotemporal features since the evolution of traffic conditions shares certain similarities in the spatial and temporal dimensions with video, speech and so forth. Wu et al. extracted spatial and temporal features by a CNN and LSTM, respectively and predicted traffic volume combining these two approaches [39]. The results showed that the convolutional LSTM structure can provide a potential alternative for traffic predictions. Wang et al. proposed an eRCNN model that integrated spatiotemporal features and then trained the recurrent CNN by reducing feedback predictive errors [40]. Yu et al. depicted the traffic network as a grid-based diagram, with each grid representing the traffic speed in a certain location [41]. Then, SRCNs that integrated the CNN with LSTM were utilized to forecast the speed of a large-scale network and the results outperformed other deep learning approaches in terms of the mean absolute percentage error (MAPE) and root mean squared error (RMSE).

In summary, since a wide spectrum of short-term prediction models have been applied in traffic forecasting, selecting the appropriate modeling approach for a specific circumstance is crucial for generating accurate and reliable prediction results. Among all the parametric and nonparametric approaches proposed for short-term traffic forecasting, deep learning methods have emerged as the alternative with the most potential due to their remarkable abilities for addressing nonlinear and high-dimensional problems. However, few prediction methods can achieve satisfactory accuracy under the conditions of structural missing data. Conventional techniques that compensate for incomplete data rely on filling in missing values with reasonable replacements before feeding the data into the prediction model [42]. Our method represents a new concept for addressing structural missing data problems. Moreover, because the inherent spatiotemporal characteristics of traffic flow affect the predictive performance to a great extent, various studies have concentrated on incorporating temporal and spatial dependencies into the modeling phase. In this paper, we propose the STCA to extract critical road sections that have the most impact and predictive power for future traffic states and future traffic conditions of the overall large-scale network are predicted based on the CRS-ConvLSTM. Essentially, the model employs partial roads to forecast the traffic states of the entire network, which is distinct from most existing prediction approaches. Furthermore, the impact of structural missing data on the performance of the model is explored.

## 3. Methodology

The study focuses on utilizing data from critical road sections to predict future traffic conditions of an overall urban transportation network. Specifically, the critical road sections are identified by a spatiotemporal correlation algorithm that simultaneously presents the spatial and temporal features of the traffic network. In this paper, a ConvLSTM NN that combines a CNN and LSTM is used to predict the future short-term traffic speed of the network.

### 3.1. Identifying Critical Road Sections by Spatiotemporal Features

#### 3.1.1. Constructing Spatial Weights Matrix

A spatial weights matrix represents the spatial dependency among road sections in a traffic network. According to graph theory [43], a traffic network can be abstracted to a directed graph G = (N, L) composed of N nodes and L links. Certain nodes of the traffic network are not directly connected through a path road. According to Tobler’s First Law [44], near traffic flows are more closely related than distant flows [45]. To exploit the static and physical properties of the transportation network, we construct a *k*-order spatial weights matrix through the topological adjacency relationships between road sections. If edges *i* and *j* are connected directly or indirectly, we call these two edges the “*k*th order neighbors” and their adjacency relationship can be expressed as Equation (1):

(1) $$e_{ij}^{\left(k\right)}=\{\begin{array}{ccc} 1, & when & adjacent\\ 0, & others & \end{array}$$

The weights $e_{ij}^{\left(k\right)}$ are nonzero only if the edges *i* and *j* are “*k*th order neighbors” and compose the square *N* × *N* spatial weights matrix $E_{k},$ which represents the *k*-order spatial relationship. For example, *k* = 1 means two road sections are directly connected; *k =* 2 means there is one road section between these two road sections; *k =* 3 means there are two road sections between these two road sections and so on. Then, the spatial weights matrix $E_{k}$ is normalized by a line to represent the impact between road sections, namely $\sum e_{ij}^{\left(k\right)}=1$. With these rules, we can construct the spatial weights matrices for the first-order and second-order adjacency matrix. Equal weights are assigned to the *k*th order neighbors. The spatial weights matrix *E* can be defined as the sum of the *k*th spatial weights matrix $E_{k}$, which is shown in Equation (2):

(2) $$E=\sum_{k=1}^{K}E_{k}$$

where *K* is the highest order of the spatial weights matrix.

#### 3.1.2. Measuring Spatiotemporal Similarity among road Sections

Since the current states of a road section have predictive power on the future conditions of adjacent roads, the first step is to measure the spatiotemporal correlations among road sections. Several approaches, including the Manhattan distance, the Euclidean distance and so forth, are suitable to calculating the correlation [46]. Allowing for the condition that the similarities are measured under a given time lag, the correlation distance, which is derived from Pearson’s correlation, is adopted as a potential method of measuring the dependence of the two time series. Suppose that there are two finite and equidistant series of *X* and *Y*; the correlation distance between these two series is given by Equation (3):

(3) D(X,Y)=1−Cor(X,Y)

where Cor(X,Y) is Pearson’s correlation between the series *X* and *Y*. Cor(X,Y) is calculated by Equation (4):

(4) $$Cor\left(X,Y\right)=\frac{\sum \left(X-\mu_{X}\right)\left(Y-\mu_{Y}\right)}{\sigma_{X}\sigma_{Y}}$$

where $\mu_{X}$ and $\mu_{Y}$ are the mean values of the series *X* and *Y*, respectively and $\sigma_{X}$ and $\sigma_{Y}$ are the variances of the series *X* and *Y*, respectively.

The formulas listed above represent the basic expression of the correlation distance. To comprehensively consider the spatial correlation of the road network with the temporal correlation, a spatial weights matrix is introduced as a spatial indicator to improve the initial correlation distance. Let $x_{i}^{t}$ represent the traffic speed in the road section *i* at time *t*, where $x_{i}^{t}=\left(x_{i}^{1},x_{i}^{2},\ldots ,x_{i}^{T}\right)$. Then, the integrated speed values of the adjacent road sections can be defined as $RX_{i}^{t}$*,* which is calculated by Equation (5):

(5) $$RX_{i}^{t}=\sum_{j=1}^{R}e_{ij}x_{j}^{t}$$

where *i* ∈ [1, *R*] is the number of the road sections, *t* ∈ [1, *T*] is the time interval and $e_{ij}$ is the spatial weight from spatial weight matrix *E*.

Then, the correlation between the speeds of road section *i* and its adjacent road sections can be calculated by Equation (6):

(6) $$Cor_{i}\left(s\right)=\frac{\sum_{t=1}^{T}\left(x_{i}^{t}-\bar{x_{i}}\right)\left(RX_{i}^{t+s}-\bar{RX_{i}}\right)}{\sqrt{\sum_{t=1}^{T}{\left(x_{i}^{t}-\bar{x_{i}}\right)}^{2}}\sqrt{\sum_{t=1}^{T}(RX_{i}^{t+s}-\bar{RX_{i})}}}$$

where *s* = (1, 2, …, *S*), (*s* > 0) is the time lag between the speeds of road section *i* and its adjacent road sections, $\bar{x_{i}}$ is the mean value of the traffic speed $x_{i}^{t}$ in road section *i* during a time duration of *T*, $\bar{RX_{i}}$ is the mean value of the integrated speed $RX_{i}^{t}$ and the other variables are similarly defined as in Equation (5).

$Cor_{i}\left(s\right)$ measures the impact that the traffic conditions of road section *i* exert on the future state of its adjacent road sections. A greater value of $Cor_{i}\left(s\right)$ correlates to a greater influence, whereas the correlation distance $D_{i}\left(s\right)$ is the inverse (Equation (7)):

(7) $$D_{i}\left(s\right)=1-Cor_{i}\left(s\right)$$

where $D_{i}\left(s\right)$ stands for the correlation distance between road section *i* and its adjacent road sections under a time delay of *s*.

#### 3.1.3. Recognizing and Extracting Critical Road Sections

The correlation distance provides a criterion to measure the spatiotemporal similarity among road sections. In this paper, if the correlation distance of a road section is smaller than that of the other sections, we call it a critical road section, which means that it has a powerful impact on the future traffic conditions of its adjacent road sections. To extract critical road sections of the traffic network, we need to rank the forces of all road sections based on correlation distances under every different delay.

In the area of multiattribute decision analysis (MADA), several methods are commonly used to comprehensively consider the trade-offs between multiple indicators, including the preference ranking organization method for enrichment evaluation (PROMETHEE), elimination and choice expressing reality (ELECTRE), analytic hierarchy process (AHP), order of preference by similarity to ideal solution (TOPSIS) and so forth. [47]. Among all these methods, TOPSIS has been effectively used for solving many different selection/ranking problems since it is a comprehensible, intuitive tool and easy to perform. Applying TOPSIS to rank the impacts of road sections can integrate correlation distances under different time lags into a rational composite index, which is called the integrated correlation distance. The details are as follows.

- Step 1: Create an evaluation matrix.

As mentioned above, we shall regard the correlation distance matrix (CDM) with elements $D_{i}\left(s\right)$ as the evaluation matrix. The CDM consists of *R* alternatives and *S* attributes that represent *R* road sections and *S* time lags respectively; hence, $\mathrm{CMD}={\left(D_{i}\left(s\right)\right)}_{S\times R}$.

- Step 2: Calculate the weights under every time lag.

Different weights are assigned to different time lags with the consideration that closer time intervals have more similar traffic states. Thus, the weights under time lag *s* are calculated using the Euclidean distance as shown in Equation (8):

(8) $$Ed\left(s\right)=\frac{\sum_{t=1}^{T-s}\sqrt{\sum_{i=1}^{R}{\left(x_{i}^{t+s}-x_{i}^{t}\right)}^{2}}}{T-s}$$

where $x_{i}^{t}$ is the traffic speed of road section *i* at time *t* and the other variables are similar to that of Equation (6). A smaller value of Ed(s) means the traffic states are more similar. However, a larger weight should be assigned to a more similar traffic state; therefore, the Ed(s) is reversed and scaled to a range of 0 to 1 by Equation (9):

(9) $$Ed^{\prime }\left(s\right)=1-\frac{Ed\left(s\right)-min\left(Ed\right)}{max\left(Ed\right)-min\left(Ed\right)}$$

where *min(Ed)* and *max(Ed)* denote the minimum and maximum of the Euclidean distance set, respectively.

- Step 3: Determine the ideal worst solution $A^{-}$ and the ideal best solution $A^{+}$.

The correlation distance is varied with the time lag; therefore, $A^{-}$ and $A^{+}$ need to change over the time lag and they are defined as Equations (10) and (11), respectively:

(10) $$A^{-}\left(s\right)=\{minD_{i}\left(s\right)|s\in \left(1,2,\ldots ,S\right),1\leq i\leq R\}\;$$

(11) $$A^{+}\left(s\right)=\left\{maxD_{i}\left(s\right)|s\in \left(1,2,\ldots ,S\right),1\leq i\leq R\right\}$$

where $A^{-}\left(s\right)$ and $A^{+}\left(s\right)$ are the ideal worst and best solution, respectively, under time lag *s* and the other variables are similar to that of Equations (7)–(9).

- Step 4: Calculate the distance between $D_{i}\left(s\right)$ and the ideal worst and best solutions.

The weighted Euclidean distance is applied to calculate the distance between $D_{i}\left(s\right)$ *a*nd the ideal worst and the best solution. The weights are involved to identify different degrees of impact under each time lag, which can be determined by Equations (8) and (9). The distance between road section *i* and the worst solution $A^{-}\left(s\right)$ is calculated by Equation (12):

(12) $$D_{i}^{-}=\sqrt{\sum_{s=1}^{l}Ed^{\prime }\left(s\right)\cdot {\left(D_{i}\left(s\right)-A^{-}\left(s\right)\right)}^{2}},\;i=1,2,3,\ldots ,R$$

The distance between the correlation distance of road section *i* and the worst solution $A{\left(s\right)}^{+}$ is determined by Equation (13):

(13) $$D_{i}^{+}=\sqrt{\sum_{s=1}^{l}Ed^{\prime }\left(s\right)\cdot {\left(D_{i}\left(s\right)-A^{+}\left(s\right)\right)}^{2}},\;i=1,2,3,\ldots ,R$$

where $D_{i}^{-}$ and $D_{i}^{+}$ are the weighted Euclidean distances between the correlation distance of road section *i* and the worst and best solution, respectively. Other variables are similarly defined as in Equations (7)–(11).

- Step 5: Calculate the similarity to the worst condition.

The similarity to the worst condition measures the degree of impact under all delays between road section *i* and its adjacent road section, which can be calculated by Equation (14):

(14) $$C_{i}=\frac{D_{i}^{-}}{D_{i}^{-}+D_{i}^{+}},0\leq C_{i}\leq 1,i=1,2,3,\ldots ,R$$

where $C_{i}$ is the similarity to the worst condition and *i* stands for the number of the road section, which is the integrated correlation distance. Obviously, $C_{i}=1$ means that the integrated correlation distance has the best condition and $C_{i}=0$ means that it has the worst condition.

- Step 6: Rank the impact and identify the critical road sections according to $C_{i}$(i=1,2,3,…,R).

$C_{i}$ measures the degree of impact between road section *i* and its adjacent road sections. A greater value of $C_{i}$ means that the traffic status of road section *i* has a greater impact on the future conditions of its adjacent road sections. Thus, the impact is ordered by the value of $C_{i}$. Furthermore, we extract a number of road sections by the percentage of α according to their orders. Then, these roads are regard as critical road sections. α is defined as extracting rate and it actually presents the number of critical road sections. Distinguished by the spatiotemporal features of traffic flow, the critical road sections can be characterized and thus used to predict the traffic conditions of the overall network. Thus, partial information can be used to predict global variables.

- Step 7: Split the time period and integrate the dataset.

Due to the unbalanced distribution of living areas and working areas, the traffic tide phenomenon (TTP) frequently occurs in large cities [48]. To distinguish the deviation that the TTP introduces to the spatiotemporal characteristics of the network, the change of critical road sections must be divided into multiple periods of time in accordance with traffic pattern (i.e., peak period or ordinary period). As a result, distinct sets of critical road sections are observed at each time period such that the number of roads remains the same, while the elements are different. In addition, the data of every time period are integrated into one dataset, which represents the critical road section dataset of the whole day. The integrated dataset will be used to train the prediction model and forecast future traffic conditions of the overall network.

### 3.2. Predicting the Short-Term Traffic State Using ConvLSTM NN

In this paper, the deep learning approach ConvLSTM NN is used as the prediction method. The ConvLSTM NN presents the advantages of the CNN and LSTM methods and can simultaneously extract spatial and temporal features.

#### 3.2.1. Reshaping Training Data to a Spatiotemporal Matrix

Traffic information generated by the floating car with a GPS device consists of both spatial and temporal features, which should work in conjunction to predict future traffic states of the network. Assume that $x_{it}$ represents the average speed on road section *i* at time *t*. Then, the spatiotemporal matrix is established with the elements of $x_{it},$ which represents the average speed on road section *i* at time *t*. In addition, the row and column of the matrix are spatially and temporally associated, respectively. As a result, the daily traffic states can be mathematically transformed into a spatiotemporal matrix with *T* rows and *R* columns, which can be denoted as Equation (15):

(15) $$M=\left[\begin{array}{cccc} x_{11}, & x_{12}, & \cdots & x_{1R}\\ x_{21,} & x_{22}, & \cdots & x_{2R}\\ \vdots & \vdots & & \vdots \\ x_{T1}, & x_{T2}, & \cdots & x_{TR} \end{array}\right]$$

where *T* is the length of time intervals and *R* is the number of road sections.

Along the time dimension (i.e., columns of the matrix), the time range that usually extends from the beginning to the end of the day is divided into narrow intervals of 2–15 min. Additionally, the speeds are also aggregated and averaged over 2–15 min in coordination with the time intervals. The length of the interval is determined primarily based on the sampling intervals and the data update frequency. Additionally, for real-time forecasting that focuses on the traffic evolution from 15 to 40 min, intervals that are too narrow or wide are inappropriate in practice.

**Figure 1:**
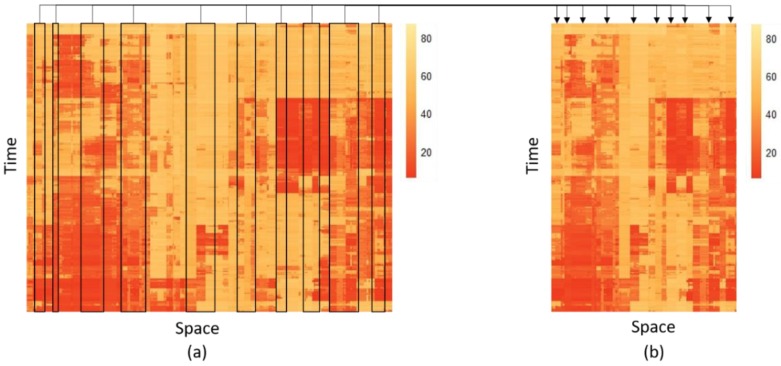
The process of shaping spatiotemporal matrix of critical road section: (**a**) A spatiotemporal matrix of overall network and (**b**) A spatiotemporal matrix of critical road sections.

Along the spatial dimension (i.e., rows of the matrix), the speeds of each road section are arranged as a sequence representing the traffic state of the network during a certain time period. Notably, for our model, the spatiotemporal matrix of critical road sections and the overall network have the same temporal dimension but a different spatial dimension since the critical road sections are extracted from the latter in the light of the spatiotemporal features. Thus, to ensure the consistency of the two types of matrices in space, the sequence of the roads will not be disrupted. In simple terms, we draw the speeds of critical road sections from the original matrix and then merge these columns together into a matrix of critical road sections. Figure 1 displays two heat plots of the traffic speed. The process simply illustrates how to generate the spatiotemporal matrix of critical road sections to form as large an overall network matrix as possible to ensure the spatial characteristics.

#### 3.2.2. Capturing Spatial Features via a CNN

**Figure 2:**
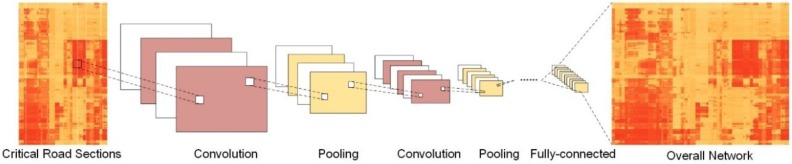
The structure of the convolutional neural networks (CNN).

The CNN has been successfully applied to traffic predictions because of its great potential to extract features using multiple layers. A typical CNN mainly consists of multiple convolution layers and pooling layers. The former contributes to mining spatial dependencies of road sections since every layer retrieves a distinct feature using different filters while the latter assists in reducing the number of parameters required for training the CNN under the premise of ensuring prediction accuracy. Because the input for a CNN can be intuitively regarded as an image in which each pixel value is associated with one type of traffic state during a certain time, a 2D CNN is naturally utilized to abstract spatial features between road sections. Figure 2 illustrates the structure of a CNN, including the input layer, convolution layer, pooling layer, fully connected layer and output layer. Each part plays a unique and vital role for a CNN and the details are briefly explained below.

Suppose that we need to predict the future traffic speed of the network $V^{t+a}={\left\{x_{i}^{t+a}\right\}}_{i=1}^{R}$, where *a* is the prediction horizon and *R* is the number of road sections. The input of the CNN is the historical traffic speed of critical road sections {$U^{t-n},U^{t-1},U^{t}$}, where $U^{t-n}={\left\{x_{i}^{t-n}\right\}}_{i=1}^{P_{\alpha }}$ represents the traffic state of critical road sections at time (*t−n)*, *n* is the look back step and $P_{\alpha }$ is the number of critical road sections. Then, the spatial features of input are captured by convolutional and pooling layers. Let $O_{r}^{l}$ be the output of *l*th convolutional and pooling layers with *r* filters and the weights and bias of lth layers be ($W_{r}^{l},b_{r}^{l}$). Then, the $O_{r}^{l}$ can be calculated by Equation (16):

(16) $$O_{r}^{l}=pool\left(f\left(\sum_{r}W_{r}^{l}*O_{r}^{l-1}+b_{r}^{l}\right)\right)$$

where $O_{r}^{l-1}$ represents the output of the previous layer, in which $O_{r}^{1}$ represents the input layer; *f* is a nonlinear activation function; and *pool* denotes the pooling procedure.

#### 3.2.3. Capturing Temporal Features Via the LSTM

**Figure 3:**
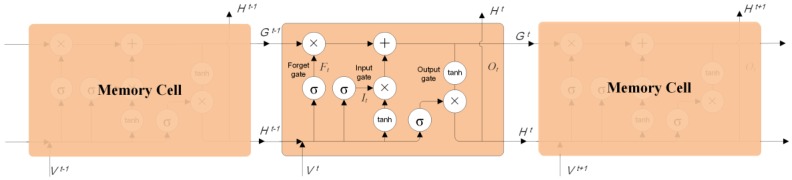
The architecture of long short-term memory neural network (LSTM NN).

Intuitively, traffic states at each moment have a strict sequential relationship in the temporal dimension rather than being isolated from each other, which is especially suitable for RNNs to capture the temporal evolution of traffic flow. However, it is difficult for traditional RNNs to capture the temporal dependency if two time intervals are remote. Thus, an LSTM, which is a specific form of a RNN, is proposed to tackle these issues by adding memory cells in hidden layers. As shown in Figure 3, four main parts (input gate, neuron with a self-recurrent connection, forget gate and output gate) collaborate to alleviate the problems of traditional RNNs caused by the gradient vanish and explosion problems.

In our model, next to the CNN, the LSTM naturally takes the output of the CNN $V^{t}={\left\{x_{i}^{t}\right\}}_{i=1}^{R}$ as its input to predict the future traffic states, namely, its output $H_{t}={\left\{h_{i}^{t}\right\}}_{i=1}^{q}$, where *q* is the number of hidden units of the output layer. For memory cell, the input state is $G_{t-1}$ while the output is $G_{t}$. Meanwhile, the states of input, forget and output gates are $I_{t},F_{t}\;\mathrm{and}\;O_{t}$, respectively. Clearly, due to some relatively simple linear processes (i.e., plus or scalar product), a long-term state can be reserved by $G_{t-1}$. However, $H_{t-1}$ represents the state of last memory cell. Through the comprehensive operation of the three gates, long-term and current states exert effect on future memory cells simultaneously. The temporal features can be iteratively calculated by Equations (17)–(22):

(17) $$\mathrm{Input}\;\mathrm{gate}:\;I_{t}=\sigma \left(W_{v}^{i}V^{t}+W_{h}^{i}H^{t-1}+b_{i}\right),$$

(18) $$\mathrm{Forget}\;\mathrm{gate}:\;F_{t}=\sigma \left(W_{v}^{f}V^{t}+W_{h}^{f}H^{t-1}+b_{f}\right),$$

(19) $$\mathrm{Output}\;\mathrm{gate}:\;O_{t}=\sigma \left(W_{v}^{O}V^{t}+W_{h}^{o}H^{t-1}+b_{o}\right),$$

(20) $$\mathrm{Cell}\;\mathrm{input}:\;G_{t-1}=tanh\left(W_{v}^{c}V^{t}+W_{h}^{c}H^{t-1}+b_{c}\right),$$

(21) $$\mathrm{Cell}\;\mathrm{output}:\;G_{t}=I_{t}⊙G_{t}+F_{t}⊙G_{t-1},$$

(22) $$\mathrm{Hidden}\;\mathrm{layer}\;\mathrm{output}:\;H_{t}=O_{t}⊙\tan h\left(G_{t}\right),$$

where the weight matrix W and bias vector *b* are constructed to connect the input layer, output layer and memory cell, ⊙ denotes the scalar product of two vectors and σ(·) represents the standard logistics sigmoid function defined in Equation (23):

(23) $$\sigma \left(x\right)=\frac{1}{1+e^{-x}}$$

#### 3.2.4. Training with the ConvLSTM NN

Integrated with the advantages of the CNN and LSTM methods, the ConvLSTM NN is utilized to predict future traffic states by sufficiently exploiting the spatiotemporal characteristics of the data. Eventually, a fully connected layer is employed to predict the future speed by taking the output of the LSTM as the input. The future speed can be calculated by Equation (24):

(24) $$Y^{t+1}=W_{y}H_{t}+b_{y}$$

where $W_{y}$ and $b_{y}$ are the weight and bias related to the hidden layer. Conclusively, the model is trained from end to end and the values of $Y^{t+1}$ are prediction results that represent the output of the entire mode. Several hyperparameters within the model will be set and elaborated in the experiment section. Notably, the input size will vary as the number of critical road sections changes due to different extracting rates α; hence, several other hyperparameters will also change.

## 4. Empirical Study in Beijing Network

### 4.1. Description of Data Used

To valid the efficiency of our methodology, data collected by a taxi equipped with GPS devices from 1 June 2015 to 13 October 2015 (92 working days) in Beijing, the capital of China, are utilized to train the ConvLSTM NN model and predict the future traffic speed of network. The updating frequency of the data is 2 min and the time period from 6:00:00 to 23:00:00 is considered for the high travel demand period, which has been repeatedly observed. Accounting for the condition that the traffic state varies with every time interval, we can observe 511 traffic states per day.

**Figure 4:**
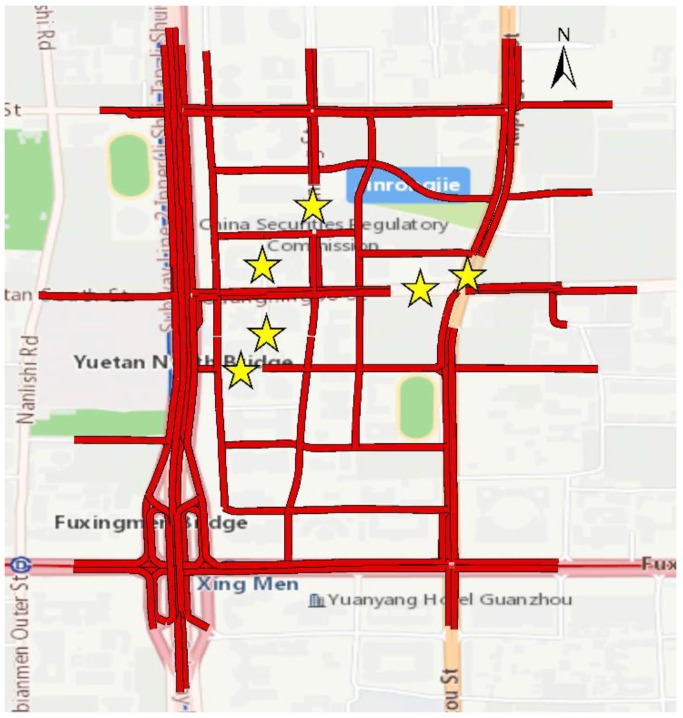
Layout of the sub-transportation network in Beijing.

In our paper, a sub-transportation network near West Second Ring Road is selected as the research objective, as shown in Figure 4. The network consists of 278 road sections, including several types of road hierarchies, such as freeways, arteries, secondary roads and collectors. The total length of all the roads is approximately 24.53 km and the network covers an approximate 0.6-km^2^ area. Two thirds of the data (61 days) are regarded as training data, while the remainder (31 days) compose the testing data. Additionally, the structural missing data marked by pentagram can be obviously observed.

### 4.2. Critical Road Sections Identification by STCA

The spatial similarity of traffic flow can be easily obtained according to the topology of the network, which will be viewed as the static properties of the traffic flow. The *k*-order spatial weights matrix is constructed to describe the topology of the traffic network. The spatial influence is measured by calculating the Euclidean distance between different road sections as shown in Equation (25):

(25) $$SP\left(k\right)=\frac{\sqrt{\sum_{t=1}^{T}\left(x_{i}^{t}-x_{i+k}^{t}\right))}}{T}$$

where SP(k) is the Euclidean distance that measures the spatial correlation between roads, *k* denotes *k*-order, *T* is the total time period and $x_{i}^{t}$ represents the speed of road section *i* at time *t*.

**Figure 5:**
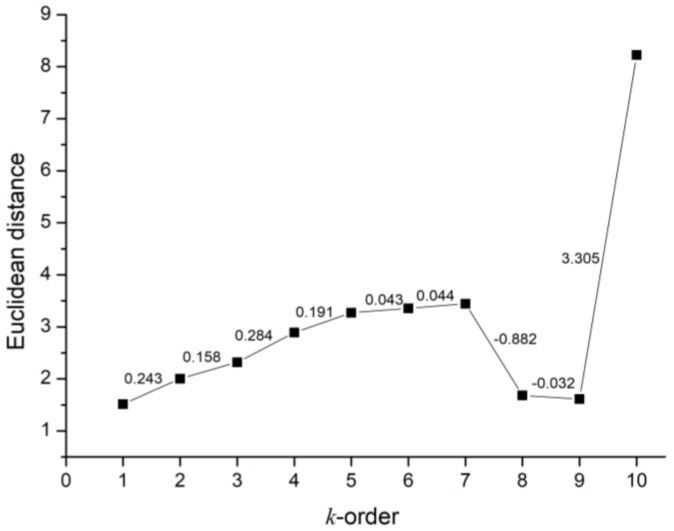
Influence between roads in the case of different *k*-order.

Figure 5 depicts obviously positive correlation between the Euclidean distance and the *k* value when *k* is less than 7. It is obvious that if *k* is bigger than 7, distant roads have weak impact on the given road. And from Figure 5, we can find that the variation between 5th and 6th order is less than previous values. Therefore, we take 5-order neighbors as adjacent road sections. Based on these results, we can conclude that only the nearby roads contribute to the current road section traffic speed while the remote ones have little influence. Moreover, a sensitivity analysis is also conducted to measure the impact of different spatial weights matrices to the predictive performance.

**Figure 6:**
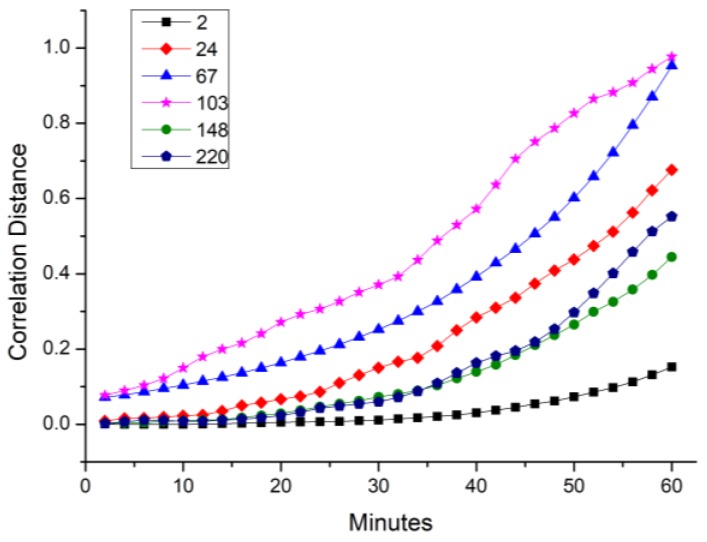
Trend of correlation distance along with the time lag.

To capture the stochastic and time-varying dynamic characteristics, the spatiotemporal features of the traffic flow should be mined by the spatial and temporal correlations. Considering the traffic flow is commonly repeated every working day [49], we average the speeds of all 92 days, which means an entry of the average speed matrix represents the average speed along 92 days in a certain road section at a moment. It can be seen that the mean of the speed is gradually tending to convergence with the increasing of the days. For most roads, the mean of the speeds becomes steady around 60 days. We take the average of 92 days to make sure as much as speeds becomes steady. In this way, we can possibly avoid special situations and extract the most universal critical road sections. Then the spatiotemporal features can be extracted from the average speed matrix. As previously discussed, similarities between road sections are measured by integrated correlation distances under different time lags. Figure 6 shows the spatiotemporal correlation distances of several road sections when time ranges from 2 min to 60 min, which represents 1 to 30 time delays respectively.

Figure 6 shows that the spatiotemporal correlation distance values increase as the time lag extends, which means that the states of the adjacent moments are more similar than the distant moments. In other words, the traffic states of one road have a stronger predictive power for the state of the nearby moment of the neighboring road network than for the distant moment. In addition, the curves for different roads present distinct growth trends, which characterize various temporal features.

Another important factor that should be taken into account for such predictions is the influence of TTP. The temporal features of traffic flow vary dynamically over time, which leads to the transition of the critical road sections. For example, one road section may play a relatively considerable role in the morning peak time rather than the evening peak time. By analyzing the evolution of traffic flow and using previous empirical knowledge [50], one day is divided into four periods in accordance with different traffic patterns: the morning peak period (6:00:00–10:00:00) (MPP), the daytime ordinary period (10:00:00–17:00:00) (DOP), the evening peak period (17:00:00–21:00:00) (EPP) and the evening ordinary period (21:00:00–23:00:00) (EOP).

**Figure 7:**
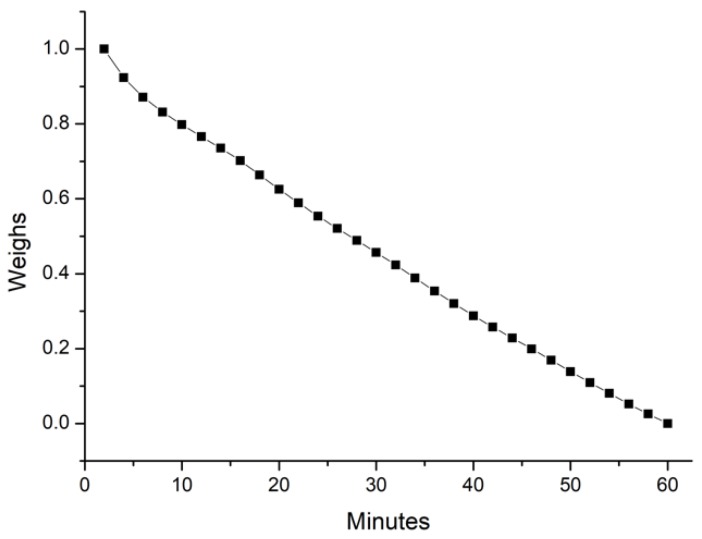
Trend of weights along with the time lag.

Subsequently, TOPSIS is employed to measure the degree of predictive power among road sections during each time period and then extract critical road sections according to the integrated correlation distances. First, the evaluation matrix of each period is constructed. Since the length of each period is different, the size of the CDM varies based on the context of each period as shown in Equation (26):

(26) $$CMD=\{\begin{array}{lll} {\left(D_{i}(s)\right)}_{120\times 278}, & for & MPP\\ {\left(D_{i}(s)\right)}_{210\times 278}, & for & DOP\\ {\left(D_{i}(s)\right)}_{120\times 278}, & for & EPP\\ {\left(D_{i}(s)\right)}_{61\times 278}, & for & EOP \end{array}$$

where 278 is the total number of the road sections. Then, the weights used to evaluate the impact of time lags ranging from 1 to 30 time steps are calculated by Equations (8) and (9). Figure 7 clearly shows that the interaction between two traffic states weakens as the minute increases, which is why we pay more attention to proximate traffic states when ranking the influence of roads.

The ideal worst and best solutions are the worst and the best values of the correlation distance under each time lag, respectively. In addition, the integrated correlation distance $C_{i}$ that measures the influence of the road on its adjacent road sections is calculated by the weighted Euclidean distance. When correlation distances corresponding to all time lags are taken into account, the spatiotemporal characteristics of the road sections will be reflected comprehensively.

**Figure 8:**
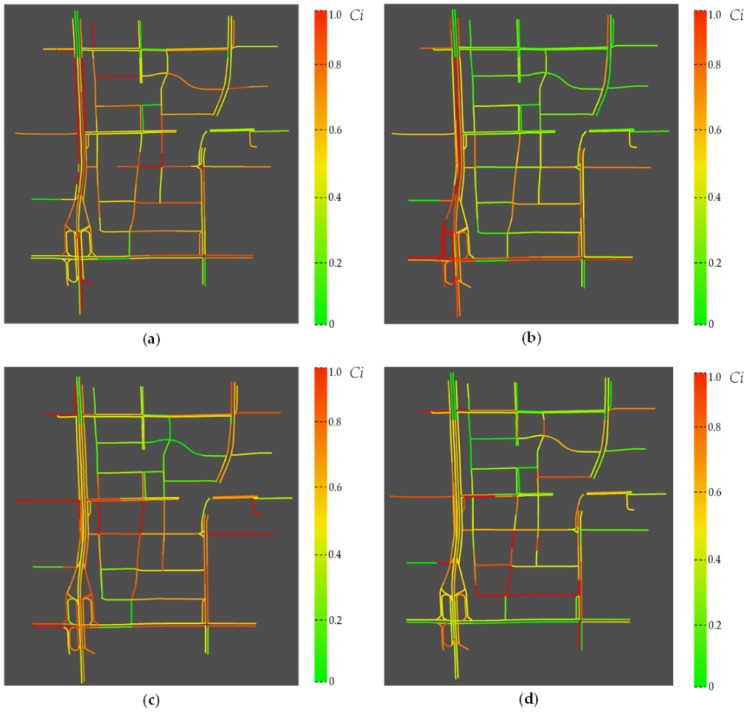
Distribution of critical road sections in the: (**a**) Morning Peak Period; (**b**) Daytime Ordinary Period; (**c**) Evening Peak Period; and (**d**) Evening Ordinary Period.

Eventually, the impact of roads is ranked according to the integrated correlation distance. Notably, to measure the performance under different road extracting rates α (i.e., the proportion of critical road sections to all roads), the number of critical road sections that are input to the prediction model varies. Then, the number of critical road sections corresponding to α is set to β. The correspondence of the extracting rate and the number of critical road sections is listed in Table 1. For example, α=0.70 means that we will take 195 roads as critical road sections and subsequently use them to predict the traffic states of 278 roads. Figure 8 shows the degree of impact of all roads at different time periods. The red line indicates that the road section has a greater influence on the future traffic state of adjacent roads in the MPP, DOP, EPP or EOP. The distribution of the red line indicates that the critical road sections vary with time, which verifies the effect of the TTP on the critical degree of the road sections. From Figure 8, we can find that roads on the left side (i.e., the West Second Ring Road) appears to be critical all the periods. The reason is that these roads are expressways which undertake major traffic flow in the network. Another interesting fact is that roads in the central of the network has more critical degrees during peak hours than ordinary hours. It is probably because that compared with expressway (i.e., the West Second Ring Road), urban roads are affected by traffic signals and they show more various spatiotemporal features. Therefore, more roads are needed to predict the traffic states of nearby road sections.

### 4.3. Training with Critical Road Sections based on the ConvLSTM NN

#### 4.3.1. Generating Input Data

In a traffic network, nearby road sections are more relative that distant ones. In order to retain the spatial correlation between adjacent roads, it is necessary to arrange adjacent roads together. An effective method is to straighten consecutive roads into a line and then construct a spatiotemporal state matrix. As stated in Ma et al. (2017) [36], a simple traffic network, such as an expressway or ring road, can be easily straightened to generate a straightforward matrix. However, complete spatial dependencies cannot be retained when reshaping the road sections of the complicated network into a straight line. To alleviate the negative effect caused by the loss of spatial relations, the network is segmented into successive routes and the roads are numbered along the route. Moreover, the CNN can capture spatial features from local connections despite the complex and network-wide dependencies of traffic speeds. After reshaping the speed of all road sections into a spatiotemporal matrix, we obtain 92 matrices and each represents the daily traffic state and are regarded as the labels of the prediction model. Using the same approach, spatiotemporal matrices that denote the traffic states of critical road sections can be generated and regarded as the input of the model.

#### 4.3.2. Tuning up with the ConvLSTM

Without a general regulation for constructing the structure of DL NNs, the selection of hyperparameters mainly depends on expert experience. In addition, compared with the prevalent CNN or LSTM NN, the spatial dimensions of the inputs and outputs in our CRS-ConvLSTM are unequal. In this section, we take 195 critical road sections (i.e., extracting rate α=0.70) as an example to explain the procedure for constructing the prediction model.

In our model, the length of the time steps is set to 15, which means that 30 min of historical data are utilized to predict the traffic state of the next 2 min. For a 2D CNN structure, a 5-layer fully convoluted structure is used so that the depth of the CNN is neither too large nor too small. Next, a 2-layer LSTM structure is employed to capture temporal features. The size of the convolutional filters and max-poolings refers to the experiments of AlexNet [51] and LeNet [52], which both made remarkable achievements in the development of CNNs. The details of the parameters are listed in Table 2. The dimension of the input layer (1, 195, 15) is determined by the size of the input data shape, where the first number denotes the channel of the input data is 1, the second is the number of critical road sections at extracting rate of 0.70 and the third represents the input time horizon, which is 15 time steps. The parameters of convolutional layer indicate the number and the size of the filters. For example, the convolutional layer of layer 1 has 16 filters and each size of them is (3, 3). With the size of (2, 2), the pooling layers shrink the output of each convolutional layer. The output dimensions of CNN in layer 5 are (256, 7, 1), which are then flattened into a vector with a dimension of 1792. Before feeding the data into two layers of LSTM, the vector is transformed with a dimension of 278 through a fully-connected layer. The dimension of the output layer is 278, which represents the total number of all the road sections.

The ADADELTA is chosen as the optimizer because of its dynamic adaptability over time [53]. The mean squared error (MSE) is selected as the loss function and 20% of the training data are extracted as the validation dataset. A batch-normalization layer is used to accelerate the training process [54]. Additionally, the dropout layer and early stopping are used to prevent overfitting [55].

## 5. Results and Comparison

### 5.1. Performance under Different Spatial Weight Matrices

In our paper, we propose a spatial weight matrix to capture the topology features of the network. In the previous section (i.e., 4.2), we have qualitatively described the influence between the road and its k-order road sections. And we can conclude that it is better to set k less than 7. Here, we adopted root mean square error (RMSE) and root mean squared error proportional (RMSEP) to evaluate the performance of the model in the case of different spatial weight matrices; the relevant formulas are show in Equations (27) and (28):

(27) $$\mathrm{RMSE}=\frac{1}{n_{0.7}}\sqrt{n_{0.7}\sum_{i=1}^{N_{0.7}}{\left({\hat{y}}_{i}-y_{i}\right)}^{2}}$$

(28) $$\mathrm{RMSEP}=\frac{100}{{\hat{y}}^{ave}}\sqrt{\frac{1}{n_{0.7}}\sum_{i=1}^{N_{0.7}}{\left({\hat{y}}_{i}-y_{i}\right)}^{2}}$$

where ${\hat{y}}_{i}$ is the ith ground truth value, $y_{i}$ is the *i*th predicted value and ${\hat{y}}^{ave}$ is the average of the ground truth values. The value of $n_{0.7}$ denotes the number of critical road sections at the extracting rate α=0.70 and $N_{0.7}$ is the total number of traffic states.

What to be noted is that we extract 195 critical road sections (i.e., extracting rate α=0.70) as input. The results under different spatial weight matrices are listed in Table 3. It is obvious that the model achieves the best when *k* is 5. Consequently, we set *k* to 5 which means we regard at most 5-order neighbors as adjacent road sections.

### 5.2. Performance between Several DL Algorithms

In order to test the performance of our CRS-ConvLSTM model, four popular deep learning-based algorithms are selected for comparison: ANN, CNN, LSTM and SAE. The ANN adopts a plain and shallow structure (Table A1) to process multidimensional and nonlinear problems. Additionally, the structures of the CNN (Table A2) and LSTM (Table A3) refer to the first part and latter part of the CRS-ConvLSTM configuration, respectively. The parameters of the SAE (Table A4) are set according to [31]. Using the same approach as the CRS-ConvLSTM implementation, 30 min of historic traffic speed for 195 critical road sections is used as the input to predict the overall traffic states after 2 min. The RMSE and RMSEP are employed to evaluate the performance of all the models.

**Figure 9:**
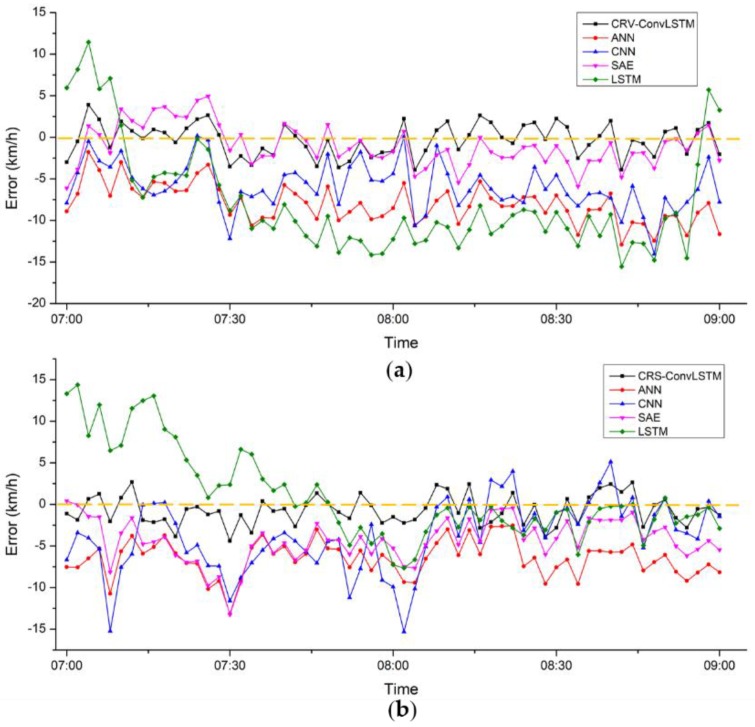
Errors between the ground truth and the estimated value on: (**a**) 3 August 2015 and (**b**) 17 August 2015.

Table 4 presents the quantitative results of the CRS-ConvLSTM, ANN, CNN and LSTM and shows that the CRS-ConvLSTM outperforms the other models, thus indicating that our CRS-ConvLSTM model can precisely mine the spatiotemporal features of the data and generate relatively accurate predictions. Among the rival algorithms, the LSTM has the best performance, which is likely because the temporal features of the time-series data are essentially prominent. The results of the ANN and SAE demonstrate that these two models fail to extract spatiotemporal characteristics that have a vital impact on prediction. Figure 9 depicts the error between the ground truth and the estimated values under different models. It can be found that the error varies with the time and day, however the error of the CRS-ConvLSTM is closer to zero most of the time.

### 5.3. Performance under Different Extracting Rates

To explore the effect of the extracting rate α on the prediction performance, we further train and test the model based on a different number of critical road sections according to Table 1. The RMSEs and RMSEPs in the context of different extracting rates α are listed in Table 5 and the results show that in the range of 0.6 to 0.9, the performance of the model is slightly superior to that of the overall prediction model (i.e., α=1.0). Here, we define a drop rate to measures the decline of the accuracy compared with the overall prediction model as shown in Equation (29):

(29) $$\mathrm{drop}\;\mathrm{rate}=\;\frac{Index_{1}-Index_{2}}{Index_{1}}$$

For drop rate in Table 5, $Index_{1}$ denotes the error index (i.e., RMSE or RMSEP) at the case of the overall prediction model and $Index_{2}$ presents the RMSE or RMSEP at the extracting rate of α.

**Figure 10:**
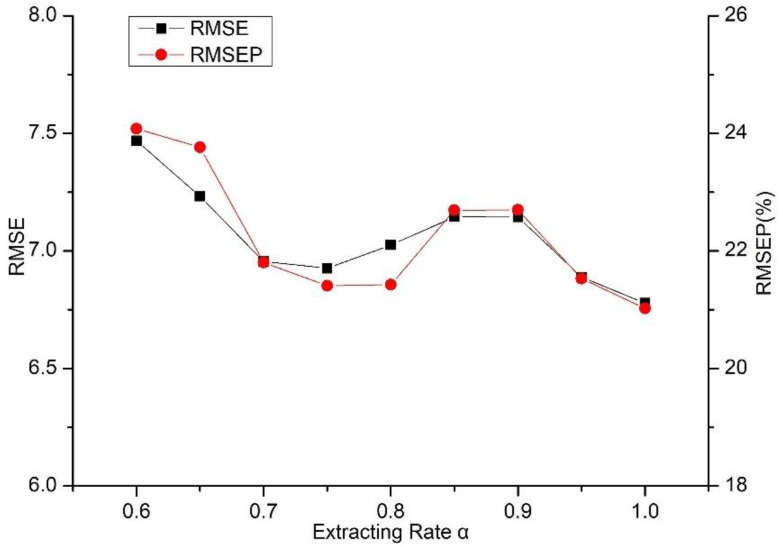
Trend of prediction accuracy under different extracting rate.

The decline of the accuracy compared with the overall prediction model is ranging from 1.638% to 10.180% for the RMSE and 1.841% to 14.537% for the RMSEP. In general, the decrease in accuracy is reasonable and within the acceptable limits, which demonstrates the validity and generalizability of the approach and indicates that certain road sections account for a lower contribution toward the prediction. Among all the extracting rates at less than 1.0, the value of 0.95 achieves the optimal result, which is primarily because the most minor road sections are removed. Additionally, when the extracting rate reaches a value of 0.65, the predictive performance gradually tends to become unstable, which is likely because too many roads are omitted. Figure 10 shows the trend of the prediction accuracy, which generally declines as the extracting rate decreases. It is interesting that we can discovery a bump up around 0.85, which can also be observed in [56].They utilized SVD-combined tensor decomposition to remedy incomplete traffic data. When filling up the fiber-like missing data (i.e., structural missing data), the RMSE and the missing rate is not a simple linear relationship and a valley is presented around 40%. Specifically, the RMSE decreases with the increase of missing rate at the first. However, when missing rate is beyond 40%, the RMSE increases with the rise of missing rate. The same trend can be witnessed when extracting rate is lower than 0.85 in our paper. One of the reasons is that recovering structural missing data is much more difficult than those of random missing [57]. It is also probably because of the distribution of critical road sections.

### 5.4. Performance for Stochastic and Extreme Cases

In the practical situation, corrupted or missing data generally occur because of monitoring equipment failure, extreme weather, data transmission errors and so forth and such data weaken the effectiveness of the prediction model or even disable the model [58]. As stated in [59], data input failure can be classified into three categories: (a) incidental (random) failures, mainly due to temporary detection or communication failures; (b) structural failures, caused by long-term equipment failures; and (c) intrinsic failures, derived from noise, bias and other inherent deviations of detection devices. For the FCD used in the research, these three types of input failures may occur simultaneously. In a network, a low traffic volume or signal obstruction caused by very large buildings may result in unreliable data in certain roads, structural failures will be the primary concern in this paper. Different from traditional methods that restore missing data by imputation methods before prediction [60], we predict the traffic conditions of the roads that suffer from structural missing data based on their adjacent critical road sections.

For the purpose of testing the performance of our model under random structural missing data, we stochastically extract a part of the road sections at the rate of α, where the value remains the same as previously mentioned. First, we assign each road section a random value that meets the uniform distribution with minimum 0 and maximum 1. Then, we sort the random values in an ascending order and extract the top road sections at the rate of α. In order to eliminate the impact of randomness, we performed 10 groups of experiments in stochastic case under each extracting rate and the final result was the average over 10 experiments.

**Figure 11:**
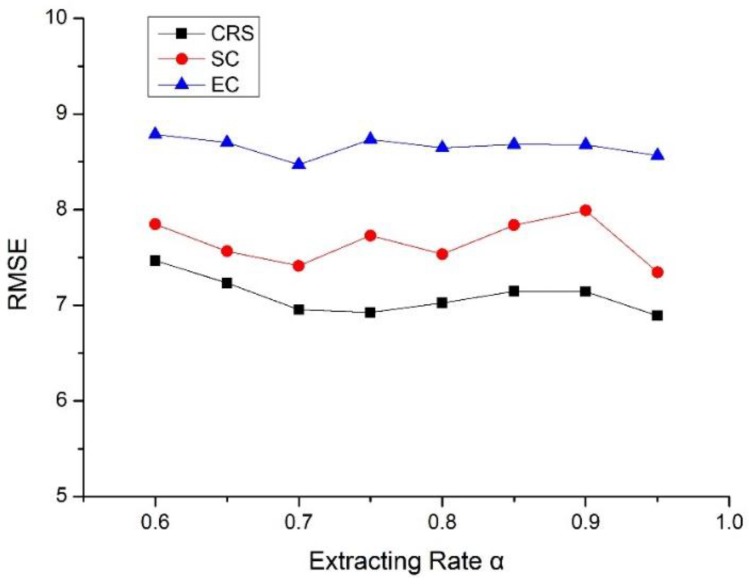
Comparison between critical road sections (CRS) case, stochastic case (SC) and extreme case (EC).

An extreme case is also involved so that the most critical road sections with greater values of $C_{i}$ are removed. Table 5 demonstrates the performance of stochastic and extreme case. The drop rate measures the gap between contrastive scenarios and the case of critical road sections. That is, for drop rate in Table 6, $Index_{1}$ of Equation (28) denotes the error index (i.e., RMSE) at the case of critical road sections and the $Index_{2}$ indicates the RMSE in the case of stochastic or extreme case at the same extracting rate of α (α < 1). It can be found that all the prediction accuracies under random missing data are inferior to the prediction performance of critical road sections the same as in the extreme case, while the performance is much worse due to the absence of the most valuable spatiotemporal characteristics. The reason that the margin of values is different for stochastic and extreme case is that the stochastic case remains more partial spatiotemporal characteristics while the extreme lost the most. Figure 11 depicts the gap among critical road sections, stochastic case and extreme case. It is obvious that predicting with the data of critical road sections performs best in all extracting rate, followed by stochastic case and extreme case.

However, although the proposed approach seems to be ineffective under stochastic and extreme cases, the problems caused by structural missing data are indeed alleviated, especially in the case of critical road sections, thus validating the effectiveness of our CRS-ConvLSTM. Moreover, the results present a meaningful and worthy instruction for traffic management that more monitoring devices should be allocated along critical road sections to improve the level of prediction.

## 6. Summary and Conclusions

Structural missing data usually have a massive negative effect on short-term traffic state predictions. In this paper, we propose a ConvLSTM traffic speed prediction method to alleviate the problem caused by structural missing data. First, the spatiotemporal features are extracted to measure the impact of road sections on adjacent roads. Spatial weights matrices are constructed to represent the topology of the network and the correlation distance is adopted to calculate the degree of impact between a road section and its local network. Then, correlation distances are integrated by TOPSIS to rank the critical degree of all roads. Thus, critical road sections can be distinguished according to their order and subsequently extracted as the input of the prediction model at different extracting rates. In the prediction section, the proposed ConvLSTM NN is employed to forecast future traffic states of the overall network based on the speeds of critical road sections.

To demonstrate the validation of the proposed model, a group of empirical experiments are performed using the traffic speed collected at 2 min intervals from a Beijing transportation network with 278 links. A sensitivity analysis about the different spatial weight matrices is conducted to prove that the availability of 5-order spatial weight matrix. Compared with the ANN, CNN, LSTM and SAE, our CRS-ConvLSTM achieves an acceptable accuracy with RMSE is of 6.955 at the extracting rate of 0.7. Furthermore, the prediction accuracy under different extracting rates is tested. The results show that in general, the RMSE increases as the extracting rate decreases and when the top 95% of road sections are used as input, we can acquire the optimal result. A bump up around 0.85 is probably because of the difficulty of recovering structural missing data and the distribution of the critical road sections. However, when the α value is lower than 0.65, the efficiency of the model gradually becomes unstable and unreliable. Consequently, the prediction accuracy is slightly affected when we omit part of the noncritical road sections and an excessive reduction of input data is not appropriate either.

Subsequently, a situation of stochastic and extreme structural missing data in a real scenario is simulated by extracting certain roads at rates ranging from 0.60 to 0.95. All the results in the context of random and extreme cases are inferior to those of critical road sections. Although obvious regularities are not observed for the RMSEs of the model under different extracting rates, the critical road sections undoubtedly have more prominent predictive power. Moreover, the validity of the spatiotemporal correlation algorithm has also been confirmed.

In summary, we tested the performance of the proposed in a large-scale network, which proved the availability of the proposed method. In practical implementation, considering that we adopted deep learning approaches, at least 3 months historical traffic data is necessary to train and test the prediction model. Before prediction, the spatiotemporal features should be extracted to draw the critical road sections out of the overall network. Once the structure of the model is determined and the parameters is trained by historical data, short-term prediction can be conducted based on the current states of the critical road sections. Thus, online prediction of the overall traffic network can be performed by states of critical road sections and the trained model.

For future studies and applications, the quality of data should be addressed since random missing values affect the prediction accuracy. The imputation method such tensor decomposition is emerging as a potential approach to remedy missing traffic data. Also, the hierarchy, length and other inherent attributes of the road should be taken into account when extracting spatial correlations between roads. Additionally, since weather, temperature, air quality and other external factors affect travel demand, these factors can be comprehensively considered to improve prediction accuracy. Another strategy for improving prediction accuracy is the inclusion of more detection devices at critical road sections to achieve a more reliable prediction.

## Figures and Tables

**Table 1:** Correspondence of extracting rate and the number of critical road sections.

Parameters	Values								
Extracting rate α	0.60	0.65	0.70	0.75	0.80	0.85	0.90	0.95	1.00
Number of roads β	167	181	195	209	222	236	250	264	278

**Table 2:** Hyperparameters of the hybrid convolutional long short-term memory neural network model based on critical road sections (CRS-ConvLSTM) when α=0.70.

Layer	Name	Parameters	Dimensions
**0**	**Input**	**--**	**(1, 195, 15)**
1	Convolution	(16, 3, 3)	(16, 195, 15)
	Pooling	(2, 2)	(16, 98, 8)
	Activation(relu)	--	--
	Batch-normalization	--	--
2	Convolution	(32, 3, 3)	(32, 98, 8)
	Pooling	(2, 2)	(32, 49, 4)
	Activation(relu)	--	--
	Batch-normalization	--	--
3	Convolution	(64, 3, 3)	(64, 49, 4)
	Pooling	(2, 2)	(64, 25, 2)
	Activation(relu)	--	--
	Batch-normalization	--	--
4	Convolution	(128, 3, 3)	(128, 25, 2)
	Pooling	(2, 2)	(128, 13, 1)
	Activation(relu)	--	--
	Batch-normalization	--	--
5	Convolution	(256, 3, 3)	(256, 13, 1)
	Pooling	(2, 2)	(256, 7, 1)
	Activation(relu)	--	--
	Batch-normalization	--	--
6	Flatten	--	(1792, )
7	Fully connected	278	(278, )
8	LSTM	200	--
	Activation(tanh)	--	--
9	LSTM	200	--
	Activation(tanh)	--	--
10	Fully connected	278	(278, )

**Table 3:** Performance in the case of different value of *k.*

Index	The Value of *k*			
	3	4	5	6
RMSE	6.972	6.967	6.955	6.989
RMSEP (%)	21.933	21.963	21.802	21.832

**Table 4:** Performance of different prediction models when α=0.70.

Model	RMSE	RMSEP (%)
CRS-ConvLSTM	6.955	21.802
LSTM	7.359	22.849
CNN	7.937	26.521
SAE	8.377	26.442
ANN	9.504	37.870

**Table 5:** Performance of the CRS-ConvLSTM under different extracting rate.

α	Prediction Performance on Test Dataset			
	RMSE	Drop Rate (%)	RMSEP (%)	Drop Rate (%)
1.0	6.778	-	21.022	-
0.95	6.889	1.638	21.530	2.417
0.90	7.144	5.400	22.698	7.973
0.85	7.145	5.415	22.693	7.949
0.80	7.025	3.644	21.425	1.917
0.75	6.926	2.184	21.409	1.841
0.70	6.955	2.611	21.802	3.710
0.65	7.232	6.698	23.763	13.039
0.60	7.468	10.180	24.078	14.537

**Table 6:** Prediction results in the case of stochastic and extreme situation.

α	Prediction Performance on Test Dataset			
	Stochastic Case		Extreme Case	
	RMSE	Drop Rate (%)	RMSE	Drop Rate (%)
0.95	7.345	6.619	8.565	24.329
0.90	7.992	11.870	8.679	21.487
0.85	7.837	9.685	8.682	21.512
0.80	7.534	7.245	8.647	23.089
0.75	7.728	11.579	8.734	26.105
0.70	7.412	6.571	8.470	21.783
0.65	7.565	4.605	8.701	20.313
0.60	7.847	5.075	8.785	17.635

## Appendix A

**Table A1:** Hyperparameters of the ANN when α=0.70.

Layer	Name	Parameters	Dimensions
0	Input	--	(195 × 15, )
1	NN	128	--
	Activation(relu)	--	--
	Dropout (0.2)	--	--
2	NN	256	--
	Activation(relu)	--	--
	Dropout (0.2)	--	--
3	NN	512	--
	Activation(relu)	--	--
	Dropout (0.2)	--	--
4	Fully connected	278	(278, )

**Table A2:** Hyperparameters of the CNN when α=0.70.

Layer	Name	Parameters	Dimensions
0	Input	--	(1, 195, 15)
1	Convolution	(16, 3, 3)	(16, 195, 15)
	Pooling	(2, 2)	(16, 98, 8)
	Activation(relu)	--	--
	Batch-normalization	--	--
2	Convolution	(32, 3, 3)	(32, 98, 8)
	Pooling	(2, 2)	(32, 49, 4)
	Activation(relu)	--	--
	Batch-normalization	--	--
3	Convolution	(64, 3, 3)	(64, 49, 4)
	Pooling	(2, 2)	(64, 25, 2)
	Activation(relu)	--	--
	Batch-normalization	--	--
4	Convolution	(128, 3, 3)	(128, 25, 2)
	Pooling	(2, 2)	(128, 13, 1)
	Activation(relu)	--	--
	Batch-normalization	--	--
5	Convolution	(256, 3, 3)	(256, 13, 1)
	Pooling	(2, 2)	(256, 7, 1)
	Activation(relu)	--	--
	Batch-normalization	--	--
6	Flatten	--	(1792, )
7	Fully connected	278	(278, )

**Table A3:** Hyperparameters of the LSTM when α=0.70.

Layer	Name	Parameters	Dimensions
0	Input	--	(1, 195 × 15)
1	LSTM	200	--
	Activation(tanh)	--	--
2	LSTM	200	--
	Activation(tanh)	--	--
3	Fully connected	278	(278, )

**Table A4:** Hyperparameters of the SAE when α=0.70.

Task	Hidden Layers	Hidden Units (Bottom-Top)
30-min traffic prediction	3	[200, 200, 200]
